# Separation of Chromium (VI), Copper and Zinc: Chemistry of Transport of Metal Ions across Supported Liquid Membrane

**DOI:** 10.3390/membranes12070685

**Published:** 2022-07-01

**Authors:** Saik Su Goh, Mohd Rafatullah, Norli Ismail, Mahboob Alam, Masoom Raza Siddiqui, Eng-Keng Seow

**Affiliations:** 1 School of Industrial Technology, Universiti Sains Malaysia, Gelugor 11800, Penang, Malaysia; saiksu@student.usm.my (S.S.G.); norlii@usm.my (N.I.); 2 Division of Chemistry and Biotechnology, Dongguk University, 123, Dongdaero, Gyeongju-si 780714, Korea; mahboobchem@gmail.com; 3 Chemistry Department, College of Science, King Saud University, Riyadh 11451, Saudi Arabia; mrsiddiqui@ksu.edu.sa; 4 School of Industrial Technology, Faculty of Applied Sciences, Universiti Teknologi MARA, Shah Alam 40450, Selangor, Malaysia; ekseow@uitm.edu.my

**Keywords:** heavy metal ion, selective recovery, separation, solvent extraction, theoretical modeling

## Abstract

Prior to applying supported liquid membranes (SLM) with strip dispersion for separation of chromium (VI), copper and zinc, suitable chemical settings were determined through solvent extraction and stripping studies. More than 90% of copper and zinc could be simultaneously extracted with at least 3% (*v*/*v*) di-(2-ethylhexyl)phosphoric acid (D2EHPA) at a feed equilibrium pH in the range of 3.5–5.0. For stripping, theoretical model equations derived and experimental results revealed that suitable concentrations of lower acid strength reagents can strip metals that have weaker metal-extractant bond without significantly stripping metals that have a stronger metal-extractant bond. Therefore, in a setup comprising three compartments separated by two SLM, we propose to fill the three compartments in the following order: feed—strip dispersion containing low acid strength reagent—strong acid. An organic phase with 4% (*v*/*v*) D2EHPA was used. From stripping experiments, 0.2 mol/L pH 3 citrate buffer, which resulted in the highest copper recovery (88.8%) and solution purity (99.0%), was employed as the low acid strength reagent while the strong acid consisted of 1 mol/L sulfuric acid. In 26 h, 99.1% copper was recovered by citrate buffer with 99.8% purity and 95.1% zinc was recovered by sulfuric acid with 98.4% purity. Chromium (VI), copper and zinc could be separated effectively using this separation strategy.

## 1. Introduction

Metals have many useful applications in our daily lives. However, as pollutants, metals pose risks to plants, animals, and humans. Increases in the use of metal for anthropogenic activities such as industrial, agricultural, and domestic activities in turn leads to the generation of more metal pollutants stemming from the resulting wastes [1,2], which could be in liquid form (mine waters, wastewaters from metal surface treatment processes such as electroplating and pickling, wastewaters from tanning, wood processing, inorganic pigment production) or solid form (solid residues from mineral processing, electrical and electronic waste, spent catalysts and batteries) [3,4]. However, it is not feasible to do away with the usage of metals. More metals continued to be mined and produced in order to meet demand. The depletion of high-grade metal ores and the need for managing metal pollution call for processes that can recover metals from low-grade ores and secondary sources through waste recycling [5,6].

Solvent extraction is one of the known techniques that can be used for removing, separating and concentrating metal ions in water solutions. Hydrometallurgical process, intended for recovering metals from low-grade metal ores and solid wastes, employs solvent extraction for recovering metal from relatively dilute multi-metal aqueous solutions after leaching of solids containing metals [7,8]. Solvent extraction allows for higher throughput and is capable of attaining higher purification but since the amount of solute transfer during solvent extraction is limited by the equilibrium distribution of the solute, a large solvent inventory is required, which in turn incurs a higher capital cost. Alternatively, liquid membrane, a more advance adaptation and combination of solvent extraction and membrane separation techniques, were found to be capable of recovering metal ions from dilute metal aqueous solutions. A supported liquid membrane (SLM), in particular, is advantageous over solvent extraction as it requires significantly less solvent and stages to operate. Due to the higher diffusion coefficient of solute in liquid, the transport flux of metal ions through SLM could possibly be higher than transport flux attained using solid polymeric membrane [8,9].

As with solvent extraction, selective recovery of metal ions can be achieved through use of suitable extractant and manipulating chemistry of feed and stripping phases [7,8,10]. In many SLM studies, which involved recovering copper, chelating oxime extractants were found to transport copper preferably over other metal ions [11,12,13], whereas solvating extractants such as Cyanex 923 were succesful in transporting metals that assumed metal-salt forms in aqueous feed [14,15]. Some studies employed ionic liquids with tailorable chemical structure to facilitate transport of target metal ions [16,17,18]. Feed chemistry-extractant pairing were manipulated in some cases to enable selective transport of metal ions over other metal ions present in feed [13,19,20]. In one study, the basic extractant, trioctylmethyl ammonium chloride (TOMAC), used could extract more than one type of metal present in the feed. Eventually, the coextracted metals could be separately recovered through selective stripping using stripping reagents with suitable alkalinity [21].

In this work, we performed solvent extraction and stripping studies to dertermine, and hence understand, the suitable chemical settings that can be applied to the supported liquid membrane for separation of chromium (VI), copper, and zinc ions. Instead of using different extractants for transporting different metal, we proposed utilizing selective stripping to separately recover metals that could be coextracted by a single type of extractant. To our knowledge, selective stripping using lower acid strength reagents in SLM system has yet to be reported. Important parameters affecting solvent extraction and stripping performance including extraction and stripping duration, feed composition and pH, extractant concentration, types of stripping reagent, stripping reagent concentration and organic-to-aqueous (O:A) volume ratio were studied. D2EHPA was used as an extractant while acetic acid, citric acid, and citrate buffer were used as stripping reagents. Theoretical model equations were derived to facilitate understanding on the effects of acid strength and concentration on stripping of copper and zinc. Based on the results from solvent extraction and stripping studies, suitable settings were selected and implemented on the SLM system. The recovery efficiency and the separation of the stripped copper and zinc were assessed.

## 2. Materials and Methods

### 2.1. Materials

Several work reported that wastewater from some plating and semiconductor industry contains metal in the range below 100 ppm to a few hundred ppm [22,23,24,25,26,27]. Synthetically prepared aqueous feed that contained mixture of 100 ppm of each chromium (VI), copper and zinc, which are commonly found in these industrial wastewater, were used in this study to represent the wastewater that is generated from those industries. The aqueous feed were prepared using potassium dichromate (K_2_Cr_2_O_7_), R&M Chemicals, Ever Gainful Enterprise Sdn. Bhd., Petaling Jaya, Malaysia, copper (II) sulfate pentahydrate (CuSO_4_·5H_2_O), Systerm, Classic Chemicals Sdn. Bhd., Shah Alam, Malaysia and zinc sulfate heptahydrate (ZnSO_4_·7H_2_O), Bendosen, Orioner Hightech Sdn. Bhd., Kuala Lumpur, Malaysia. D2EHPA (Sigma-Aldrich, Saint Louis, MI, USA, purity 97%) was employed as extractant that is diluted in kerosene (R&M Chemicals, Ever Gainful Enterprise Sdn. Bhd., Petaling Jaya, Malaysia) according to desired concentration. For pH adjustment purposes, 5 mol/L sodium hydroxide solution prepared by dissolving sodium hydroxide pellet (R&M Chemicals, Ever Gainful Enterprise Sdn. Bhd., Petaling Jaya, Malaysia) and 1 mol/L sulfuric acid diluted from concentrated sulfuric acid (95–97%) (QRëC, May Chemical Sdn. Bhd., Rawang, Malaysia) were used. Stripping reagents and buffers were prepared using glacial acetic acid (QRëC, May Chemical Sdn. Bhd., Rawang, Malaysia), citric acid monohydrate (Bendosen, Orioner Hightech Sdn. Bhd., Kuala Lumpur, Malaysia), sodium acetate trihydrate and anhydrous sodium sulfate (R&M Chemicals, Ever Gainful Enterprise Sdn. Bhd., Petaling Jaya, Malaysia). Deionized water (resistivity: 18.2 MΩ·cm) was used in preparing all aqueous solutions. All chemicals used were A.R. grade except mentioned otherwise and the chemicals were used without further purification.

Hydrophobic, circular, polytetrafluoroethylene (PTFE), flat sheet membrane filters with diameter of 90 mm, thickness of 200 μm, pore size of 0.22 μm and porosity above 95% (Labserv, Thermo Fisher Scientific, Shanghai, China) were used as the solid membrane support in the SLM experiment.

### 2.2. Equipment

All extraction and stripping experiments were performed using capped conical flasks shaken by an orbital shaker (IKA KS 4000 i control, IKA® Works (Asia) Sdn. Bhd., Rawang, Malaysia). pH of the aqueous solutions was measured using a bench top pH meter (Mettler Toledo EL20, Changzhou, China). Concentration of metal ions in aqueous solutions were determined using atomic absorption spectrophotometer (AAS) (Shimadzu AA-7000, Shimadzu Corporation, Kyoto, Japan). Concentration of chromium, copper and zinc ions were measured at wavelengths of 357.9 nm, 324.8 nm and 213.9 nm, respectively.

For the study on using SLM for separation of three different metals, three compartments and three stirrers made of glass were employed to minimize any chemical interactions with the feed and reagents used. The compartments and stirrers were custom-made by the glass blowing workshop under the School of Chemical Sciences, Universiti Sains Malaysia. The glass stirrers were used together with the overhead stirrers (IKA Microstar 7.5 Control, IKA®-Werke GmbH & CO. KG, Staufen, Germany) for stirring.

### 2.3. Methods

#### 2.3.1. Procedure for Study on Extraction

Extraction was carried out by placing the aqueous feed and organic phase containing D2EHPA together into a conical flask and shaking the flask at 250 rpm to enable mixing of the two phases. A shaking speed of 250 rpm was selected for all extraction and stripping process since the phases appeared to be well-mixed at this speed. Immediately after shaking, the content was transferred into a separating funnel to allow separation. The denser aqueous phase was retrieved as soon as when clear separation could be visually observed and its metal content was measured using AAS to determine the amount of metal ions extracted.

In order to establish the duration of extraction time required for both aqueous feed and organic phase to achieve equilibrium, equal amount of pH 3 aqueous feed and organic phase containing 3% (*v*/*v*) D2EHPA (O:A volume ratio = 1:1) were shaken in a conical flask for a designated duration (t = 1, 5 or 10 min). No further increase in extraction indicated that equilibrium was attained. The required time for the phases to achieve equilibrium was henceforth applied in subsequent experiments.

For studying the effect of aqueous phase equilibrium pH, equal amount of aqueous feed and organic phase containing 3% (*v*/*v*) D2EHPA (O:A ratio = 1:1) were shaken in a conical flask until equilibrium was achieved. Its pH was checked and adjusted gradually by adding small quantities of either 1 mol/L sulfuric acid or 5 mol/L sodium hydroxide until target pH, pH_eq_ was achieved at equilibrium.

Use of buffer in regulating pH during extraction is a feasible option instead of the tedious practice of gradually adjusting pH of non-buffered aqueous phase using strong acid and alkali. This also helps to minimize the change in the O:A ratio. Extraction was carried out on the aqueous feed containing 0.1 mol/L of citric acid-sodium citrate and acetic acid-sodium acetate buffers to study the effect of buffer on extraction.

On the other hand, the effect of extractant concentration on metal ions extraction was studied by varying the concentration of D2EHPA in kerosene. The organic solvent with different extractant concentrations was used to extract metal ions from acetic acid-sodium acetate buffered feed with pH_eq_ targeted at 3.60 ± 0.02.

#### 2.3.2. Procedure for Study on Stripping

As with extraction, stripping was carried out in a similar manner except that pH adjustment was not required since the pH of the stripping solutions did not change much since the stripping solutions had relatively higher H^+^ ions concentration. Organic solvent pre-loaded with metal ions were used for stripping studies. Different parameters studied include required stripping time for the phases to reach equilibrium, different types and concentration of stripping solution, and O:A volume ratio.

#### 2.3.3. Metal Ions Transport and Separation Using SLM with Strip Dispersion

**Figure 1:**
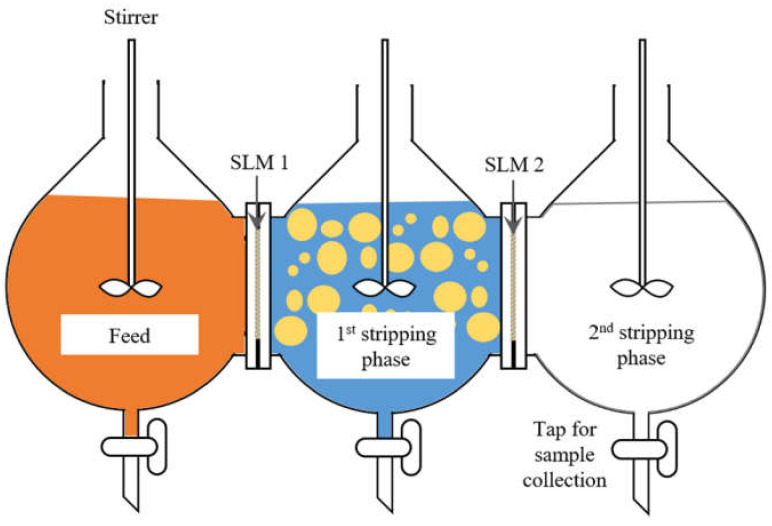
Proposed SLM setup for separation of three types of metal; feed contained 100 ppm chromium (VI), copper and zinc in 0.1 mol/L acetate buffer to maintain suitable pH, two flat sheet SLM were clamped between the feed and stripping phases compartments, first stripping phase comprised of lower acid strength reagents mixed with organic phase (D2EHPA in kerosene) to form a strip dispersion, second stripping phase consisted of strong acid.

The three compartments were assembled such as shown in Figure 1 with one sheet of PTFE membrane filter clamped securely between each compartment. The organic phase, containing a set concentration of D2EHPA in kerosene, were poured into the first stripping phase compartment until the organic liquid level were slightly in contact with the membrane filters on both sides. Since the PTFE membrane is highly hydrophobic, impregnation of the membrane filters was rapid. The membrane filters became uniformly translucent in less than five minutes. Care was taken not to add the organic phase in excess since an excess organic phase could penetrate the membrane filters easily and move into the neighboring compartments. After impregnation of the membrane filters, the feed and the second stripping phase reagent were poured into their respective compartments before filling the first stripping phase compartment. This was to further prevent any excess of organic phase flowing into the neighboring compartments. Finally, the first stripping phase compartment was filled with the designated reagent and the organic phase at a set O:A ratio to form a strip dispersion when stirred. Stirring was set to operate at 350 rpm for all three compartments. At this stirring speed, the first stripping phase appeared to be well dispersed.

When collecting samples for analysis, all of the stirrers were switched off to allow the strip dispersion to separate before retrieving the aqueous samples. Distinct separation could be observed within five minutes. Samples were collected at scheduled time intervals and their metal contents were analyzed using AAS.

### 2.4. Theory and Associated Equations

The extractant, D2EHPA, used in this study is a type of acidic extractant. D2EHPA tends to appear in dimeric form in aliphatic organic solvents and was found to extract divalent metal ions, *M*^2*+*^ according to Equation (1) [28,29,30,31]:

(1) $$M^{2+}{}_{\left(aq\right)}+\frac{2+x}{2}{\left(LH\right)}_{2}{}_{\left(org\right)}⇌ML_{2}{\left(LH\right)}_{x}{}_{\left(org\right)}+2H^{+}{}_{\left(aq\right)}$$

whereby *LH* represents D2EHPA and (*LH*)_2_ represents dimeric form of D2EHPA. Value of *x* was determined to be 2 for both extraction of copper and zinc through equilibrium slope analysis (details are provided in the Appendix A). Hence, extraction of copper and zinc can be represented by Equations (2) and (3), respectively:

(2) $$Cu^{2+}{}_{\left(aq\right)}+2{\left(LH\right)}_{2}{}_{\left(org\right)}⇌CuL_{2}{\left(LH\right)}_{2}{}_{\left(org\right)}+2H^{+}{}_{\left(aq\right)}$$

(3) $$Zn^{2+}{}_{\left(aq\right)}+2{\left(LH\right)}_{2}{}_{\left(org\right)}⇌ZnL_{2}{\left(LH\right)}_{2}{}_{\left(org\right)}+2H^{+}{}_{\left(aq\right)}$$

Percentage of metal extracted (*E*) from aqueous feed into solvent can be calculated by Equation (4) but using Equation (5) is sufficient when volume change is negligible during extraction [32].

(4) $$E\left(\%\right)=\frac{m_{M,init}-m_{M,fin}}{m_{M,init}}\times 100=\frac{{\left(\left[M^{2+}\right]V\right)}_{init}-{\left(\left[M^{2+}\right]V\right)}_{fin}}{{\left(\left[M^{2+}\right]V\right)}_{init}}\times 100$$

(5) $$E\left(\%\right)=\frac{{\left[M^{2+}\right]}_{init}-{\left[M^{2+}\right]}_{fin}}{{\left[M^{2+}\right]}_{init}}\times 100$$

whereby *m_M_* denotes mass of a metal in aqueous feed, [*M*^2*+*^] denotes concentration of metal ion and *V* denotes volume. The subscript *init* and *fin* are used to indicate before and after extraction.

For calculating percentage of metal stripped (*S*) from the loaded solvent, Equation (6) was used [32]:

(6) $$S\left(\%\right)=\frac{m_{M,strip}}{m_{M,org}}\times 100=\frac{{\left(\left[M^{2+}\right]V\right)}_{strip}}{{\left(\left[M^{2+}\right]V\right)}_{org}}\times 100$$

whereby *m_M,strip_* and *m_M,org_* corresponds to mass of metal in stripping phase after stripping and mass of metal loaded into organic phase before stripping while the subscript *strip* and *org* refer to stripping and organic phase, respectively. The concentration of metal ions loaded into the organic solvent ([*M*^2*+*^]*_org_*) can be calculated from the mass balance equations, Equations (7) and (8), that describe transfer of metal ions from an aqueous feed to an organic solvent during extraction.

(7) $$m_{M,init}-m_{M,fin}=m_{M,org}$$

(8) $${\left(\left[M^{2+}\right]V\right)}_{init}-{\left(\left[M^{2+}\right]V\right)}_{fin}={\left(\left[M^{2+}\right]V\right)}_{org}$$

In addition, percentage of metal transferred to or recovered (*R*) in stripping phase relative to initial mass of the metal present in aqueous feed (*m_M,init_*) can be calculated according to Equation (9) [33].

(9) $$R\left(\%\right)=\frac{m_{M,strip}}{m_{M,init}}\times 100=\frac{{\left(\left[M^{2+}\right]V\right)}_{strip}}{{\left(\left[M^{2+}\right]V\right)}_{init}}\times 100$$

With regards to stripping, which is the reverse of extraction, the stripping equilibrium constant of copper (*K_str,Cu_*) and zinc (*K_str,Zn_*) can be expressed as per Equations (10) and (11) [34]:

(10) $$K_{str,\;\;Cu}=\frac{1}{K_{ex,Cu}}=\frac{\left[Cu^{2+}\right]{\left[{\left(LH\right)}_{2}\right]}^{2}}{\left[CuL_{2}{\left(LH\right)}_{2}\right]{\left[H^{+}\right]}^{2}}$$

(11) $$K_{str,\;Zn}=\frac{1}{K_{ex,Zn}}=\frac{\left[Zn^{2+}\right]{\left[{\left(LH\right)}_{2}\right]}^{2}}{\left[ZnL_{2}{\left(LH\right)}_{2}\right]{\left[H^{+}\right]}^{2}}$$

whereby *K_ex,Cu_* and *K_ex,Zn_* correspond to extraction equilibrium constant for copper and zinc while [*Cu*^2*+*^] and [*Zn*^2*+*^] denote concentration of copper and zinc stripped, [(*LH*)_2_] denotes concentration of free dimeric D2EHPA that is not bound to any metal ions at stripping equilibrium, [*CuL*_2_(*LH*)_2_] and [*ZnL*_2_(*LH*)_2_] denote concentration of copper and zinc-D2EHPA complex remaining in the organic phase at stripping equilibrium and [*H^+^*] denotes concentration of hydrogen ions at stripping equilibrium. *K_ex,Cu_* and *K_ex,Zn_* were determined from the intercepts of extraction equilibria linear plots of log_10_ *D* against log_10_ [(*LH*)_2_] (details are provided in the Appendix A). *K_ex,Cu_* is 3.82 × 10^−4^ whereas *K_ex,Zn_* is 0.169.

The effect of acid strength and concentration used for stripping copper and zinc can be elucidated by rewriting *K_str,Cu_* and *K_str,Zn_* as functions of [*Cu*^2*+*^] and [*Zn*^2*+*^] stripped and other known variables. Equation (12) shows partial dissociation of weak organic acid in aqueous solution:

(12) $$HA_{\left(aq\right)}⇌H^{+}{}_{\left(aq\right)}+A^{-}{}_{\left(aq\right)}$$

Table 1 summarizes the change in organic acid, H^+^ ions and acid anions concentrations ([*HA*], [*H*^+^], [*A*^−^]) during stripping and concentrations attained after reaching stripping equilibrium. Based on Equations (2) and (3), number of mols of H^+^ ions required for stripping is twice the number of mols of each copper and zinc ions stripped. H^+^ ions will be consumed during stripping and, for stripping O:A ratio of 1:1, the amount of acid will be reduced by (2[*Cu*^2*+*^] + 2[*Zn*^2*+*^]) while (2[*Cu*^2*+*^] + 2[*Zn*^2*+*^]) of acid anions will be generated. When stripping equilibrium is reached, no further copper and zinc can be stripped. The concentration [*HA*], [*H^+^*], and [*A^-^*] that remains at stripping equilibrium can be calculated as shown in Table 1.

Therefore, acid dissociation constant (*K_a_*) can be expressed as per Equation (13)

(13) $$K_{a}=\frac{\left[H^{+}\right]\left[A^{-}\right]}{\left[HA\right]}=\frac{\left[H^{+}\right]\left(\left[H^{+}\right]+2\left[Cu^{2+}\right]+2\left[Zn^{2+}\right]\right)}{\left[HA\right]-\left[H^{+}\right]-2\left[Cu^{2+}\right]-2\left[Zn^{2+}\right]}$$

Rearranging Equation (13) and applying quadratic formula to obtain [*H^+^*] at equilibrium, [*H^+^*] can be written as a function of copper and zinc ions stripped, *K_a_* and concentration of organic acid used for stripping, [*HA*]. This is shown in Equations (14) and (15):

(14) $$\left[H^{+}\right]=\frac{-2\left[Cu^{2+}\right]-2\left[Zn^{2+}\right]-K_{a}+\sqrt{{\left(2\left[Cu^{2+}\right]+2\left[Zn^{2+}\right]+K_{a}\right)}^{2}+4K_{a}\left(\left[HA\right]-2\left[Cu^{2+}\right]-2\left[Zn^{2+}\right]\right)}}{2}$$

(15) $$\left[H^{+}\right]=-\left[Cu^{2+}\right]-\left[Zn^{2+}\right]-0.5K_{a}+0.5\sqrt{{\left(2\left[Cu^{2+}\right]+2\left[Zn^{2+}\right]+K_{a}\right)}^{2}+4K_{a}\left(\left[HA\right]-2\left[Cu^{2+}\right]-2\left[Zn^{2+}\right]\right)}$$

Concentration of metal that remains bound to D2EHPA in the organic phase at equilibrium after stripping ([*CuL*_2_(*LH*)_2_] and [*ZnL*_2_(*LH*)_2_]) can be calculated through material balance equation. For O:A ratio = 1:1, concentration of copper and zinc-D2EHPA complex at stripping equilibrium can be calculated using Equations (16) and (17):

(16) $$\left[CuL_{2}{\left(LH\right)}_{2}\right]={\left[CuL_{2}{\left(LH\right)}_{2}\right]}_{init}-\left[Cu^{2+}\right]$$

(17) $$\left[ZnL_{2}{\left(LH\right)}_{2}\right]={\left[ZnL_{2}{\left(LH\right)}_{2}\right]}_{init}-\left[Zn^{2+}\right]$$

whereby [*CuL*_2_(*LH*)_2_]*_init_* and [*ZnL*_2_(*LH*)_2_]*_init_* are initial concentration of copper and zinc-D2EHPA complex present in the organic phase before stripping. On the other hand, [*(LH)*_2_] at stripping equilibrium can be calculated by subtracting the concentration of D2EHPA that is bound to copper and zinc at stripping equilibrium from the total concentration of D2EHPA ([*LH*]*_tot_*) used as shown in Equation (18):

(18) $$[{\left(LH\right)}_{2}]=0.5\left({\left[LH\right]}_{tot}-4\left[CuL_{2}{\left(LH\right)}_{2}\right]-4\left[ZnL_{2}{\left(LH\right)}_{2}\right]\right)$$

Substituting Equations (16) and (17) into Equation (18), Equation (19) is obtained:

(19) $$[{\left(LH\right)}_{2}]=0.5{\left[LH\right]}_{tot}-2\left({\left[CuL_{2}{\left(LH\right)}_{2}\right]}_{init}-\left[Cu^{2+}\right]\right)-2\left({\left[ZnL_{2}{\left(LH\right)}_{2}\right]}_{init}-\left[Zn^{2+}\right]\right)$$

Hence, substituting Equations (15)–(17) and (19) into Equations (10) and (11), *K_str,Cu_* and *K_str,Zn_* become Equations (20) and (21). Given that *K_str,Cu_*, *K_str,Zn_*, [*LH*]*_tot_,* [*CuL*_2_*(LH)*_2_]*_init_*, [*ZnL*_2_*(LH)*_2_]*_init_*, *K_a_* and [*HA*] are known, [*Cu*^2*+*^] and [*Zn*^2*+*^] stripped can be estimated by simultaneously solving Equations (20) and (21):

(20) $$K_{str,\;\;Cu}=\frac{\left[Cu^{2+}\right]{\left[0.5{\left[LH\right]}_{tot}-2\left({\left[CuL_{2}{\left(LH\right)}_{2}\right]}_{init}-\left[Cu^{2+}\right]\right)-2\left({\left[ZnL_{2}{\left(LH\right)}_{2}\right]}_{init}-\left[Zn^{2+}\right]\right)\right]}^{2}}{\left({\left[CuL_{2}{\left(LH\right)}_{2}\right]}_{init}-\left[Cu^{2+}\right]\right){\left[-\left[Cu^{2+}\right]-\left[Zn^{2+}\right]-0.5K_{a}+0.5\sqrt{{\left(2\left[Cu^{2+}\right]+2\left[Zn^{2+}\right]+K_{a}\right)}^{2}+4K_{a}\left(\left[HA\right]-2\left[Cu^{2+}\right]-2\left[Zn^{2+}\right]\right)}\right]}^{2}}$$

(21) $$K_{str,\;Zn}=\frac{\left[Zn^{2+}\right]{\left[0.5{\left[LH\right]}_{tot}-2\left({\left[CuL_{2}{\left(LH\right)}_{2}\right]}_{init}-\left[Cu^{2+}\right]\right)-2\left({\left[ZnL_{2}{\left(LH\right)}_{2}\right]}_{init}-\left[Zn^{2+}\right]\right)\right]}^{2}}{({\left[ZnL_{2}{\left(LH\right)}_{2}\right]}_{init}-\left[Zn^{2+}\right]){\left[-\left[Cu^{2+}\right]-\left[Zn^{2+}\right]-0.5K_{a}+0.5\sqrt{{\left(2\left[Cu^{2+}\right]+2\left[Zn^{2+}\right]+K_{a}\right)}^{2}+4K_{a}\left(\left[HA\right]-2\left[Cu^{2+}\right]-2\left[Zn^{2+}\right]\right)}\right]}^{2}}$$

For calculating the average of a metal transport flux (J) across SLM after certain elapsed time, Equation (22) was used [19]:

(22) $$J\left(\mathrm{mol}/m^{2}\cdot s\right)=\frac{\Delta \left[M^{2+}\right]}{\Delta t}.\frac{V}{A}$$

whereby Δ[*M^2+^*] denotes metal concentration change in either the feed or stripping phase over elapsed time (Δ*t*), *V* denotes the aqueous volume of the phase being analyzed, and *A* denotes the effective membrane area where the transport of metal ions occurred.

## 3. Results and Discussion

### 3.1. Effect of Shaking Time on Extraction

**Figure 2:**
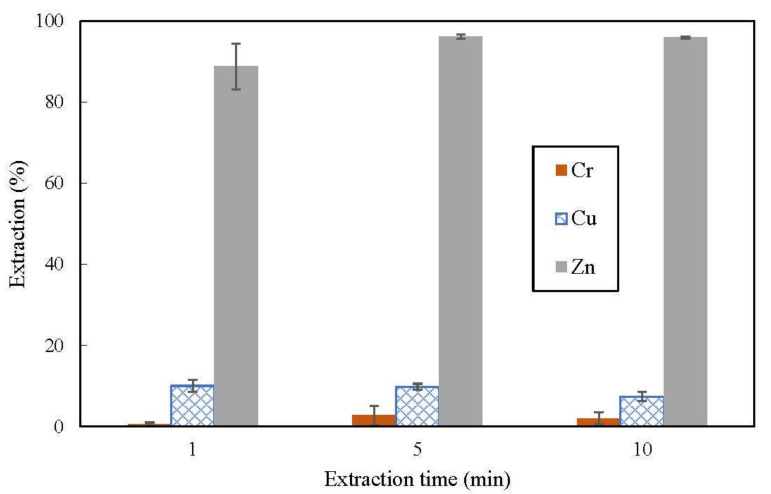
Effect of shaking time on extraction of metal ions from pH 3 feed containing 100 ppm chromium (VI), copper and zinc; organic phase consisted of 3% (*v*/*v*) D2EHPA in kerosene.

Aqueous feed containing 100 ppm each of chromium (VI), copper and zinc, with initial pH of 3, were subjected to extraction using kerosene containing 3% (*v*/*v*) D2EHPA at O:A of 1:1. Under this setting, the extraction results plotted on Figure 2 shows that D2EHPA preferably extract zinc over copper and chromium (VI). Nonetheless, extraction of zinc appeared to increase with shaking time; there were increase in zinc extraction from 1 to 5 min but there was no further increase in extraction when the phases were shaken for more than 5 min. This indicated that the phases had achieved equilibrium after 5 min of shaking at 250 rpm. The duration taken for the phases to achieve equilibrium coincides with results from other studies that used D2EHPA [35,36]. Subsequent extraction experiments were carried out with at least 5 min of shaking time.

### 3.2. Effect of pH_eq_ on Extraction

pH of feed solution has an important effect on the extent of metal extraction since the extraction reaction which occur with the use of D2EHPA, involves metal cation exchange with the hydrogen ion on the extractant [8]. During extraction, organic soluble metal-extractant complexes are formed and hydrogen ions are released into the aqueous feed as shown by Equations (2) and (3). Therefore, lower concentration of hydrogen ions present in aqueous feed or aqueous feed with higher pH will help shift extraction equilibrium forward and consequently increase extraction of metal ions.

**Figure 3:**
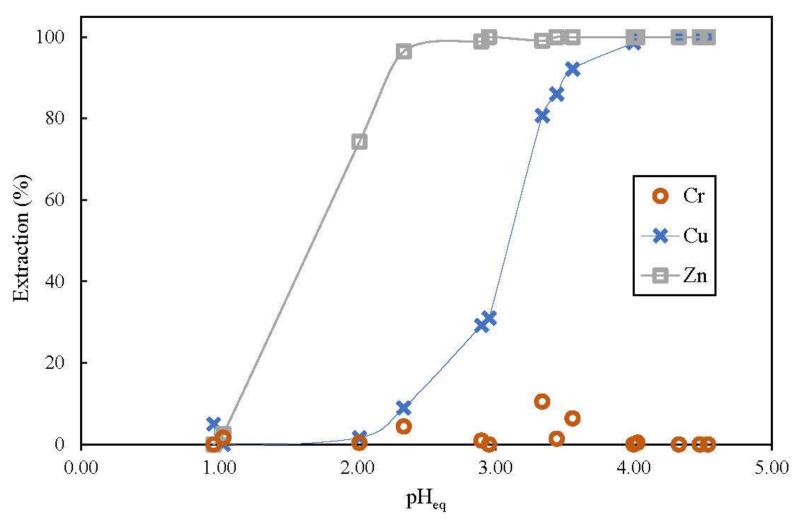
Effect of pH_eq_ on extraction of metal ions from feed containing 100 ppm chromium (VI), copper and zinc; organic phase consisted of 3% (*v*/*v*) D2EHPA in kerosene, extraction time = 5 min.

As shown in Figure 3, the extraction results for copper and zinc coincide with theoretical projection. More metal ions were extracted as pH_eq_ increased and the results reinforce that D2EHPA is a stronger zinc extractant since it was able to extract more zinc than copper at pH_eq_ below 3. As much as 74.3% of zinc could be achieved at pH_eq_ of 2 whereas extraction of copper is negligible at this pH. Extraction of copper became more significant at pH_eq_ above 3. Separation could be attained by adjusting the pH_eq_ to 2.5 or lower for applications that require only extraction of zinc whereas adjusting the pH_eq_ to 3.5 or higher allows extraction of both copper and zinc. At pH_eq_ of 4 and above, almost 100% of both copper and zinc were extracted. However, adjusting pH_eq_ to more than 4 became increasingly difficult. Emulsification was observed, the aqueous feed and solvent phase took much longer time to separate, and the aqueous feed appeared to be somewhat cloudy after extraction.

Conversely, extraction of chromium (VI) was consistently low at all pH_eq_ tested. In acidic feed, chromium (VI) was reported to usually form H_2_CrO_4_ at pH below 1 or anionic species HCrO_4_^−^ and Cr_2_O_7_^2−^ at pH 2–6 [37]. Those forms could not be extracted by D2EHPA that could only extract metal species in cationic forms. However, very low amount of chromium (VI) was sometimes extracted possibly since some chromium (VI) may had been converted into chromium (III), a cationic species extractable by D2EHPA [38].

### 3.3. Effect of Buffer in Feed on Extraction

**Figure 4:**
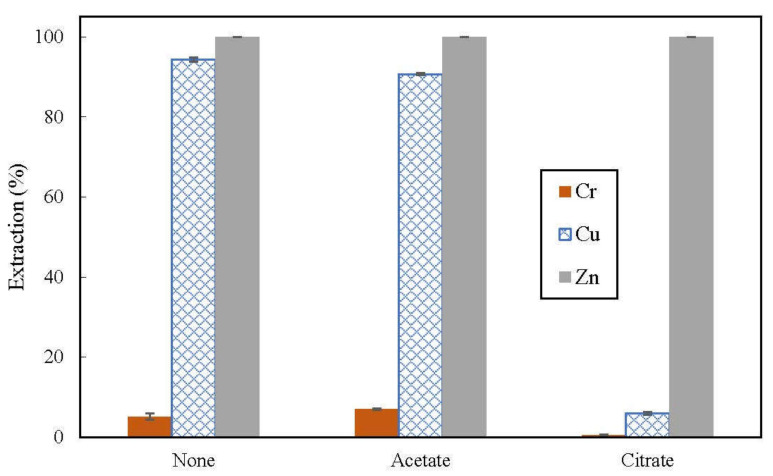
Effect of buffer on extraction of metal ions from feed containing 100 ppm chromium (VI), copper and zinc; organic phase consisted of 3% (*v*/*v*) D2EHPA in kerosene, pH_eq_ = 3.60 (±0.02), extraction time = 5 min.

Based on the study on the effect of pH_eq_ on extraction, pH_eq_ should be targeted within the range of 3.5–4.5 to achieve high extraction efficiency of both copper and zinc. In order to prevent the problems encountered during extraction at higher pH_eq_, pH_eq_ was set at 3.60 ± 0.02 for the study on effect of buffers. Citric acid-sodium citrate and acetic acid-sodium acetate buffers were selected to be included in the aqueous feed as pH_eq_ of 3.60 lies within their buffering capacity [39]. However, we found that 0.1 mol/L buffer systems could not sufficiently maintain extraction pH_eq_ at 3.60 ± 0.02 for the concentration of D2EHPA used. Slight deviation in pH_eq_ required adjustment with 1 mol/L sulfuric acid or 5 mol/L sodium hydroxide. Nonetheless, pH adjustment was much less tedious compared with pH adjustment for non-buffered aqueous feed. The extraction results are presented in Figure 4.

The buffers appeared to have negligible effect on zinc extraction. Almost 100% of zinc was extracted for both non-buffered and buffered feed. In contrast, the buffers had observable effect on copper extraction. Acetate buffer had slightly lowered copper extraction from 94.3% to 90.6% while the citrate buffer suppressed copper extraction drastically to below 10%. At higher sodium ions concentration, which was brought about by sodium acetate in the buffer, it was reported that sodium could compete against copper for extraction [40]. Besides that, it was also reported that buffers can form a complex with metal ions [39]. Copper citrate complex that was not extractable by D2EHPA may had been formed. Extraction of chromium (VI) remained low even with the presence of buffers, indicating that the buffers did not alter chromium (VI) anions into a form extractable by D2EHPA but it is unclear whether citrate buffer may have any effect in lowering extraction of chromium (III).

Thus, using citrate buffer for controlling pH_eq_ of feed is beneficial to hinder extraction of copper and it can be applied if only extraction of zinc is desirable. If extraction of both copper and zinc is required, then using acetate buffer is more suitable instead.

### 3.4. Effect of Extractant Concentration on Extraction

**Figure 5:**
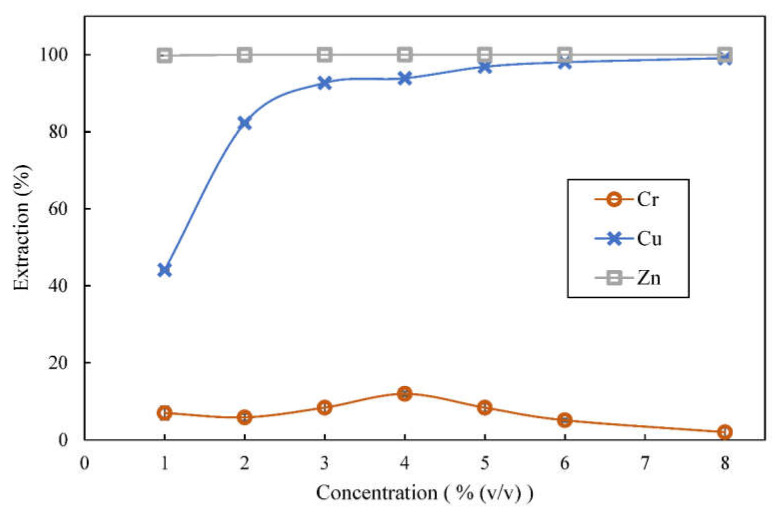
Effect of concentration of D2EHPA on extraction of metal ions from feed containing 100 ppm chromium (VI), copper and zinc in 0.1 mol/L acetate buffer; organic phase consisted of D2EHPA in kerosene, pH_eq_ = 3.60 (± 0.02), extraction time = 5 min.

With reference to Equations (2) and (3), it can be deduced that adding more extractant helps to increase extraction of metal ions by driving the reaction equilibrium forward. This can be seen from copper extraction result in Figure 5. At the same pH_eq_, copper extraction was 44.1% when 1% (*v*/*v*) D2EHPA was used and increasing concentration of D2EHPA used had increased copper extraction. Up to 99.1% of the copper was extracted when 8% (*v*/*v*) D2EHPA was used.

Interestingly, D2EHPA concentration as low as 1% (*v*/*v*) could already extract almost 100% of zinc ions and extraction of zinc remained at almost 100% when higher concentrations of D2EHPA were used, which again indicates that D2EHPA has higher affinity towards zinc than towards copper. In other works that dealt with higher feed metal concentration, higher concentration of D2EHPA was required to achieve extraction above 90%. Nevertheless, higher extractant concentration promotes coextraction of metal with similar extraction characteristics. They showed that coextraction of some other metals increased as well with increase in concentration of extractant used [35,36]. In this study, more copper was coextracted with zinc when higher concentrations of D2EHPA were used. More than 90% of copper and zinc could be extracted by using 3% (*v*/*v*) or higher concentrations of D2EHPA for extraction at O:A of 1:1 and pH_eq_ of 3.60.

If required, chromium (VI) could be subsequently removed from the feed after extraction of copper and zinc by using other suitable basic or solvating extractants such as Cyanex 923, which had been demonstrated to be effective in extracting chromium (VI) [37,41].

### 3.5. Effect of Shaking Time on Stripping

**Figure 6:**
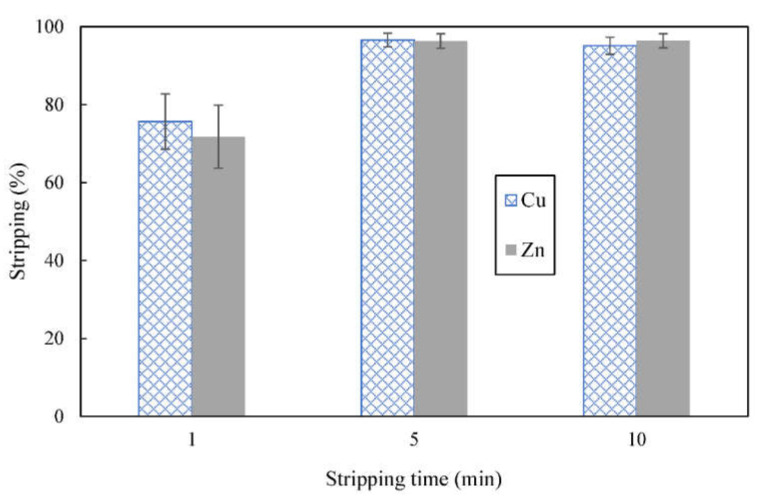
Effect of shaking time on stripping of metal ions from loaded organic phase; stripping solution consisted of 0.25 mol/L sulfuric acid, concentration of D2EHPA used was 4% (*v*/*v*).

To study the effect of shaking time on stripping, 0.25 mol/L sulfuric acid was used to strip solvent (concentration of D2EHPA = 4% (*v*/*v*)) loaded with metal ions at O:A of 1:1. Results on Figure 6 shows that stripping of copper and zinc increased when shaking time was increased from 1 to 5 min. More variability was observed at stripping time of 1 min. This was most probably due to the fact that the phases had not attained equilibrium. About 96% of each of the metals were stripped after 5 min of shaking time. Sulfuric acid is a strong acid that could completely ionize in aqueous solution and the concentration of hydrogen ions released was able to indiscriminately strip almost all of copper and zinc from the solvent. There was no further increase in stripping when the phases were shaken for 10 min. This indicated that stripping duration of 5 min, at 250 rpm shaking speed was adequate for the phases to reach equilibrium. Subsequent stripping experiments were carried out with 5 min of shaking time.

### 3.6. Use of Acetic Acid as a Stripping Reagent

**Figure 7:**
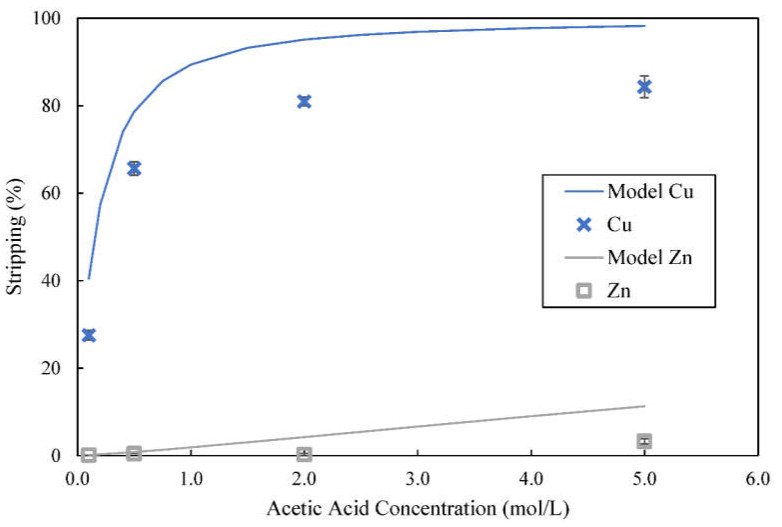
Effect of concentration of acetic acid on stripping of metal ions from loaded organic phase; concentration of D2EHPA used was 4% (*v*/*v*), stripping time = 5 min.

Stripping copper and zinc from the organic phase involves moving extraction reaction backwards to free the metal ions from D2EHPA. Higher acid concentration will increase the chemical potential difference between organic and stripping phases in terms of difference in concentration of hydrogen ions. Therefore, higher concentration of hydrogen ions in the stripping phase will promote stripping of more metal ions from acidic extractant such as D2EHPA. Based on Equations (20) and (21), the amount of copper and zinc ions stripped using different concentration of acetic acid were calculated and plotted as line graphs whereas experimentally obtained stripping results are represented by markers in Figure 7. Both calculated and experimentally obtained results showed that increasing concentration of acetic acid increased stripping of copper. With the use of 5 mol/L acetic acid for stripping at O:A volume ratio of 1:1, 84.3% of copper could be stripped from the solvent. However, there is a noticeable gap between calculated and experimentally obtained results. This gap could be due to simplified derivation of Equations (20) and (21) that did not consider activity factors.

In contrast, very low amount of zinc was stripped by acetic acid. Only 3.3% of zinc was stripped from the solvent when stripping was carried out with 5 mol/L acetic acid, which was the highest concentration used. Compared with sulfuric acid (acid dissociation constant, pK_a1_ = −3 at 25 °C) [42], acetic acid (pK_a_ = 4.7 at 25 °C) is a weaker acid that does not dissociate completely in aqueous solution [34]. Since *K_ex,Zn_* is considerably higher than *K_ex,Cu,_* D2EHPA-zinc complex formed was more stable than D2EHPA-copper complex. On the contrary, *K_str,Zn_* is much lower than *K_str,Cu_* and acetic acid was not strong enough to drive stripping of zinc from D2EHPA.

**Figure 8:**
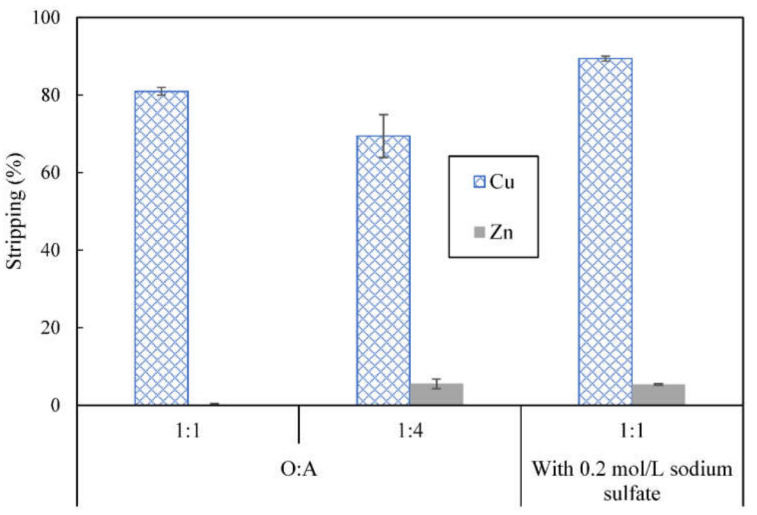
Effect of O:A ratio and addition of sodium sulfate on stripping of metal ions from loaded organic phase; all stripping solutions contained 2 mol/L acetic acid, concentration of D2EHPA used was 4% (*v*/*v*), stripping time = 5 min.

Besides studying the effect of concentration of acetic acid on stripping, the effect of O:A volume ratio used during stripping was examined as well. About 81.0% of copper and less than 1% of zinc were stripped using 2 mol/L acetic acid at O:A ratio of 1:1. Higher volume of stripping phase was used to check whether this could increase stripping but as shown in Figure 8, stripping of copper decreased to 69.4% while 5.5% of zinc was stripped instead when O:A volume ratio of 1:4 was applied. Increasing the stripping phase volume to O:A ratio of 1:4 did not increase the total amount of metal stripped.

After stripping, slight cloudiness was observed in the stripping phase, indicating that some emulsification may had occurred. Given that increasing salt concentration in stripping phase can help reduce solubility of solvent components by salting out [43], 0.2 mol/L of sodium sulfate was incorporated into the stripping phase containing 2 mol/L acetic acid. Stripping at O:A ratio of 1:1 with added sodium sulfate resulted in slightly higher stripping of copper (89.4%) and some stripping of zinc (5.4%) compared with when no sodium sulfate was used.

Using 2 mol/L acetic acid containing 0.2 mol/L sodium sulfate as the first stripping phase (O:A = 1:1), 84.4% of copper was recovered and this resulted in a copper solution with purity of 94.5%. After being subjected to the first stripping phase, 1 mol/L of sulfuric acid was used as the second stripping reagent (O:A = 1:1) to strip the remaining metals from the organic phase. Zinc recovery was 89.6% and the purity of the resulting zinc solution was 97.5%.

### 3.7. Use of Citric Acid and Citrate Buffer as Stripping Reagents

**Figure 9:**
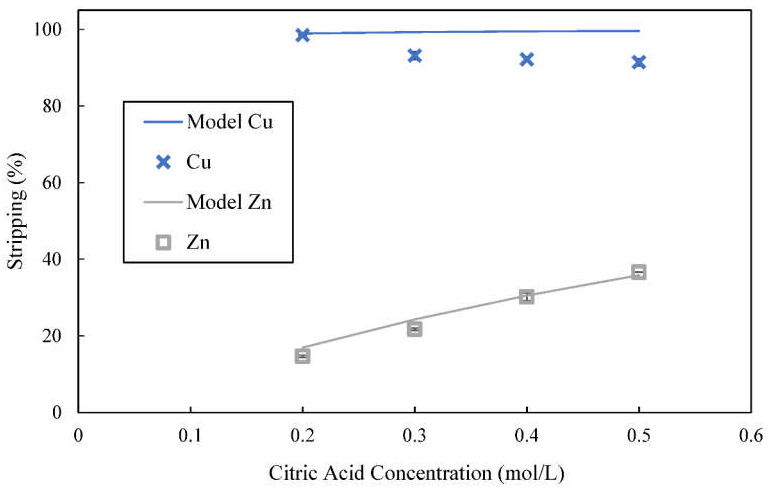
Effect of concentration of citric acid on stripping of metal ions from loaded organic phase; concentration of D2EHPA used was 4% (*v*/*v*), stripping time = 5 min.

In comparison to acetic acid, citric acid is a relatively stronger acid with pK_a1_ of 3.1 at 25 °C but is a weaker acid compared with sulfuric acid [34]. The pH of 0.5 mol/L of acetic acid was 2.45 while 0.5 mol/L of citric acid had pH of 1.55. The stripping result for varying concentration of citric acid is shown in Figure 9. Both calculated and experimentally obtained results showed that the stripping efficiency produced using citric acid is higher than the stripping efficiency obtained using acetic acid. The higher stripping efficiency achieved can be attributed to the strength of citric acid. Even the lowest concentration used, which was 0.2 mol/L of citric acid, could already strip 98.5% of the copper and 14.7% of the zinc.

The calculated amount of zinc ions stripped correspond well to the experimentally obtained results. Stripping of zinc increased considerably when higher concentrations of citric acid were used. However, Equations (20) and (21) could not account for the decline in copper stripping when higher concentrations of citric acid were used. The experimentally obtained result is unlike the result obtained with acetic acid whereby stripping of copper increased with use of higher concentrations of acetic acid for stripping. The highest concentration of citric acid used, which was 0.5 mol/L, had stripped 91.5% of copper and 36.6% of zinc from the organic phase. On the other hand, citric acid is not so strong as sulfuric acid that could strip more than 95% of both metals.

**Figure 10:**
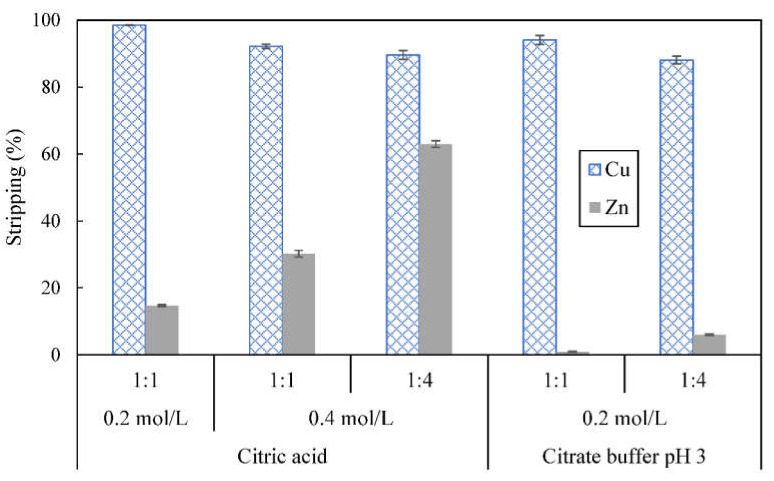
Effect of O:A ratio and citrate buffer on stripping of metal ions from loaded organic phase; concentration of D2EHPA used was 4% (*v*/*v*), stripping time = 5 min.

When studying the effect of O:A volume ratio on stripping, 0.4 mol/L of citric acid was used. Figure 10 shows that 92.2% of copper and 30.2% of zinc were stripped from the organic phase when stripping was carried out at O:A ratio of 1:1. When volume of stripping phase was increased to O:A ratio of 1:4, stripping of copper was slightly reduced to 89.6% but stripping of zinc was increased to 63.0%, more than double the quantity of zinc stripped at O:A ratio of 1:1. The effect of O:A ratio on stripping was magnified when citric acid, which is a stronger acid, was used for stripping whereas O:A ratio had less effect on stripping of zinc when acetic acid, which is a weaker acid, was used.

As opposed to stripping using acetic acid, cloudiness was not observed in the stripping phase after stripping. Low concentration of citric acid was sufficient to strip high amount of copper. The amount of copper stripped by 0.2 mol/L citric acid was higher than the amount of copper that could be stripped using 5 mol/L acetic acid. Despite that, more than 10% of zinc could be stripped along as well and this will lead to poor separation. Potentially, use of citric acid with concentration lower than 0.2 mol/L may reduce stripping of zinc.

Alternatively, citrate buffer, a reagent for stabilizing pH and controlling release of hydrogen ions, was considered for use as a stripping reagent. The lowest possible pH achievable with citrate buffer, which is pH 3, was selected for the stripping study. Stripping with 0.2 mol/L pH 3 citrate buffer at O:A ratio of 1:1 managed to strip 94.1% of copper from the organic phase as shown in Figure 10. Since 0.2 mol/L pH 3 citrate buffer comprises of about 0.116 mol/L citric acid and 0.084 mol/L of other citrate anion species, stripping was also carried out using 0.116 mol/L citric acid for comparison. About 97.8% copper and 10.0% zinc was stripped. The amount of copper stripped by 0.2 mol/L pH 3 citrate buffer was slightly lower than the amount of copper that had been stripped using 0.116 (97.8%) and 0.2 mol/L (98.5%) of citric acid but the citrate buffer used could suppress stripping of zinc to lower than 5%. When the same citrate buffer was used for stripping at O:A ratio of 1:4, stripping of copper reduced slightly further to 88.0% while 6.0% of zinc was stripped.

With 0.2 mol/L pH 3 citrate buffer as the first stripping phase (O:A = 1:1), 88.8% of copper was recovered and the resulting copper solution had purity of 99.0%. After the first stripping, the organic phase was subsequently stripped with 1 mol/L of sulfuric acid to strip the remaining metals from the organic phase. Zinc recovery was 95.1% and the purity of the resulting zinc solution was 98.3%. Compared with 2 mol/L of acetic acid containing 0.2 mol/L of sodium sulfate, 0.2 mol/L pH 3 citrate buffer resulted in fairly higher recovery of copper and better purity in the first stripping phase. Since stripping of zinc by the citrate buffer was very low, higher amount of zinc could be recovered in the second stripping phase. Overall, use of 0.2 mol/L pH 3 citrate buffer as the first stripping phase led to better separative recovery of copper and zinc.

### 3.8. Metal Ions Transport and Separation Using SLM with Strip Dispersion

Based on the results from solvent extraction studies, the following settings were applied to the SLM setup shown in Figure 1:

- First compartment contained 400 mL feed with mixture of 100 ppm each chromium (VI), copper and zinc in 0.1 mol/L acetate buffer at starting pH of 3.96 (pHeq of 3.60 could be attained when this starting feed pH was applied during solvent extraction)
- Organic phase contained 4% (*v*/*v*) D2EHPA in kerosene
- The effective membrane area was 28.3 cm^2^
- Second compartment contained 300 mL first stripping phase, 0.2 mol/L pH 3 citrate buffer, mixed with 50 mL organic phase to form a strip dispersion
- Third compartment contained 400 mL second stripping phase, which was 1 mol/L sulfuric acid

**Figure 11:**
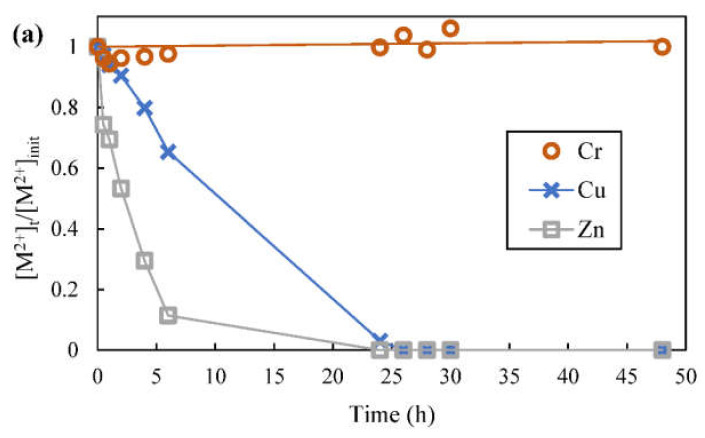

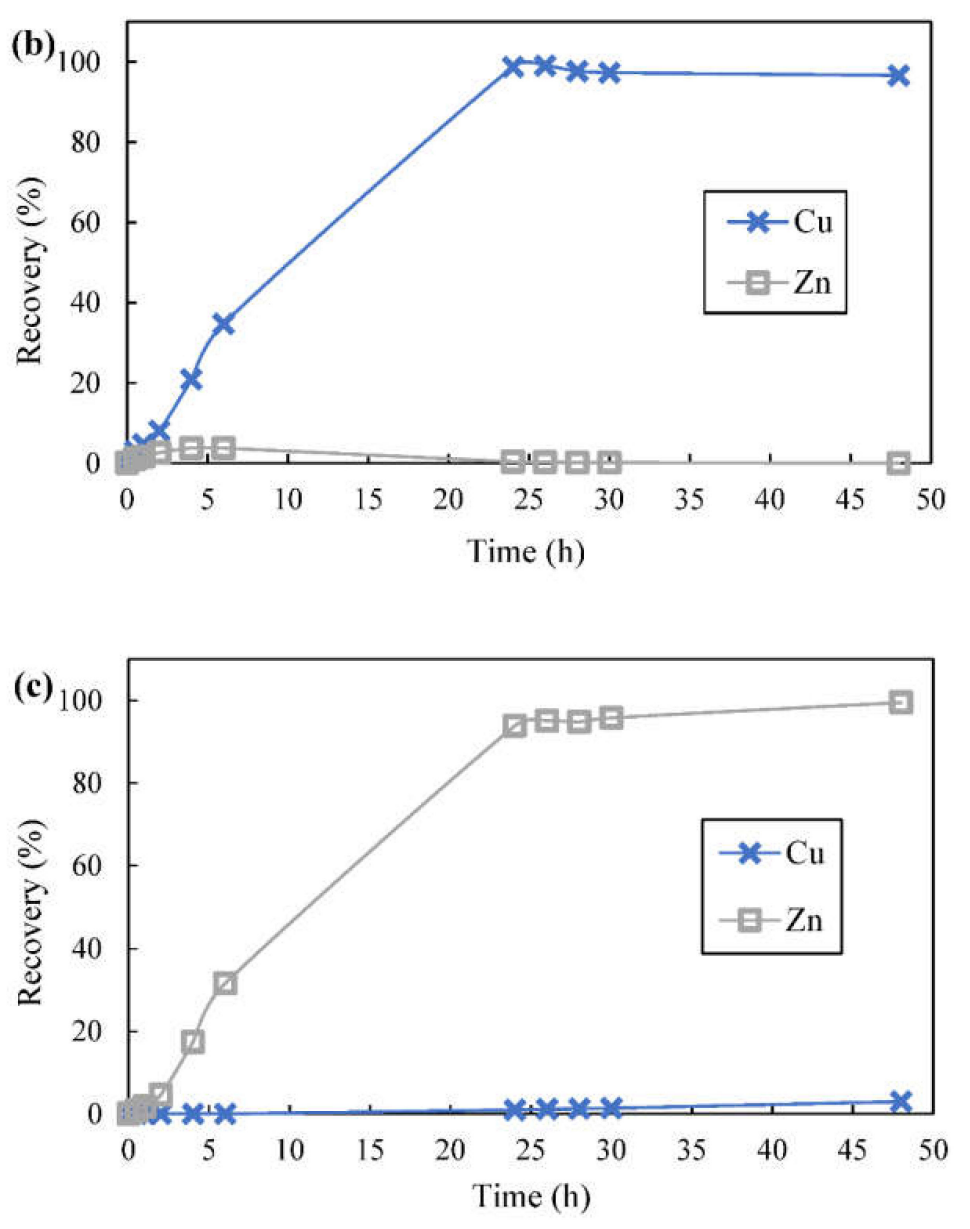
Metal ions transport and recovery using SLM; (**a**) change of metal ions concentration in feed over time, (**b**) recovery of metal ions in the first stripping phase (0.2 mol/L pH 3 citrate buffer) over time, and (**c**) recovery of metal ions in the second stripping phase (1 mol/L sulfuric acid) over time.

The metal ions transport across the SLM are detailed in Figure 11. Figure 11a shows the change in metal ions concentration in feed with respect to their initial concentrations as metal ions were transported across SLM 1 over time, whereby [M^2+^]_t_ denotes the metal ions concentration in feed after a certain elapsed time and [M^2+^]_init_ denotes the initial feed metal ions concentration. Drop in [M^2+^]_t_/[M^2+^]_init_ over time indicates a decrease in metal ions concentration due to transport of metal ions across the SLM. There was no decrease in chromium (VI) concentration as it could not be extracted and transported by D2EHPA. After 6 h, 65.3% of copper and 11.4% of zinc remained in the feed phase, indicating that 34.7% of copper and 88.6% of zinc had been transported across SLM 1. The average transport flux for the first 6 h for copper and zinc were 4.02 × 10^−6^ mol/m^2^·s and 1.06 × 10^−5^ mol/m^2^·s, respectively. Zinc was transported across SLM 1 more rapidly since D2EHPA was more preferential towards zinc as implied by solvent extraction study. After 24 h, almost all of the copper and zinc were removed from the feed.

Metal ions that were removed from the feed were recovered in the first and second stripping phase. The recovery of metal ions was calculated using Equation (9). Figure 11b shows the recovery of metal ions that pass through SLM 1 into the first stripping phase (0.2 mol/L pH 3 citrate buffer) whereas Figure 11c shows the recovery of metal ions that pass through SLM 2 into the second stripping phase (1 mol/L sulfuric acid), both over duration of 48 h. In the first stripping phase, copper recovery increased over time; close to 34.7% of copper was recovered after 6 h, which coincided with the amount of copper transported from the feed. For up to 26 h of runtime, copper recovery continued to increase until 99.1% but then decreased slightly after 26 h since a small amount of copper had moved into the second stripping phase instead. Figure 11c shows that detectable but low amounts of copper started appearing in the second stripping phase after more than 24 h and the amount copper continued to increase very slowly. After 48 h, about 3% of copper, originally present in the feed, ended up in the second stripping phase. Even though sulfuric acid in the second stripping phase was strong enough to strip both copper and zinc from the organic phase, strip dispersion in the first stripping phase had provided much higher mass transfer area for stripping of copper before the copper in the organic phase can reach the stripping interface of SLM 2. Once the copper ions were recovered in the first stripping phase, citrate buffer in the first stripping phase helped to inhibit the extraction and transport of copper ions across SLM 2 albeit transport of copper ions cannot be totally prevented.

On the other hand, Figure 11b shows that zinc was hardly recovered by citrate buffer in the first stripping phase. Zinc recovery reached 3.8% at most after 4 h of runtime and zinc content in the first stripping phase continued to drop to negligible amount after that. Based on stripping results from solvent extraction study, the citrate buffer presented mass transfer resistance towards zinc since it could not strip zinc from the organic phase, thus impeding transport of zinc ions into the first stripping phase although zinc transport flux from the feed was higher than copper transport flux. As a result of this, zinc remained in the organic phase in the strip dispersion until it could reach the SLM 2 stripping interface, whereby zinc ions could be stripped by strong sulfuric acid in the second stripping phase. This also means that the zinc transport path length is longer, and therefore recovery of zinc in the second stripping phase was initially slower than zinc transport from the feed. In the first 6 h, 88.6% of zinc had already been transported across SLM 1 but Figure 11c shows that only 31.5% of zinc was recovered in the second stripping phase at that time. After 24 h, more than 90% of zinc could be recovered and recovery of zinc reached 99.5% after 48 h.

Table 2 summarizes the metal recovery and purity of the first and second stripping phases after running the SLM for 24 to 48 h. In contrast with copper recovery, which decreased after 26 h, zinc recovery increased continuously to highest 99.5% at 48 h. Consequently, purity of copper in the first stripping phase increased over time as well due to zinc ions leaving the first stripping phase and being recovered in the second stripping phase. However, purity of zinc in the second stripping phase decreased over time instead due to transport of low amount of copper ions from the first stripping phase. Therefore, the SLM runtime can be adjusted according to the goal of treatment (such as desired purity of recovered metal solution). For instance, 26 h of SLM runtime could result in 99.1% copper and 95.1% zinc recovery with purity of 99.8% and 98.4%, respectively.

## 4. Conclusions

Solvent extraction and stripping studies demonstrated the feasibility of applying selective stripping in SLM system for the separation of chromium (VI), copper and zinc ions. The acidic extractant, D2EHPA (≥3% (*v*/*v*)) diluted with kerosene could simultaneously and rapidly extract more than 90% of copper and zinc that were in cationic forms in acetate buffer (3 ≤ pHeq ≤ 5) but could not extract chromium (VI), which does not assume cationic form in acidic solution. For selective stripping, the theoretical model equations developed could elucidate the effect of acid strength and concentration on stripping. The model corresponded with the experimental results, which show that lower acid strength reagents, acetic acid, and citrate buffer could strip large portion of copper without stripping substantial amount of zinc. Among the reagents, 0.2 mol/L pH 3 citrate buffer, which resulted in 88.8% copper recovery and 99.0% purity, was the best choice for selective stripping of copper. By applying all of the suitable chemical settings to the three compartment SLM setup, copper and zinc could be completely removed, leaving chromium (VI) in the feed compartment. Up to 99.1% copper (purity = 99.8%) could be transported to and recovered in the first stripping compartment using citrate buffer. At the same time, 95.1% zinc (purity = 98.4%) could be transported to and recovered in the second stripping compartment using 1 mol/L sulfuric acid. High metal recovery and good separation were achieved.

## Figures and Tables

**Table 1:** Change in [*HA*], [*H^+^*], and [*A^−^*] during stripping and their concentrations attained at stripping equilibrium.

Concentration (mol/L)	*HA_(aq)_*	⇌	*H^+^_(aq)_*	+	*A^−^_(aq)_*
Initial	[*HA*]		0		0
During stripping	[*HA*] − (2[*Cu*^2*+*^] + 2[*Zn*^2*+*^])		2[*Cu*^2*+*^] + 2[*Zn*^2*+*^] (Used up for stripping)		2[*Cu*^2*+*^] + 2[*Zn*^2*+*^]
Stripping equilibrium	[*HA*] − [*H^+^*] − 2[*Cu*^2*+*^] − 2[*Zn*^2*+*^]		[*H^+^*]		[*H^+^*] + 2[*Cu*^2*+*^] + 2[*Zn*^2*+*^]

**Table 2:** Metal recovery and purity of the stripping phases over time.

Time (h)	Cu in 1st Stripping Phase		Zn in 2nd Stripping Phase	
	Recovery (%)	Purity (%)	Recovery (%)	Purity (%)
24	98.8	99.7	93.8	98.7
26	99.1	99.8	95.1	98.4
28	97.7	99.8	94.8	98.3
30	97.3	99.9	95.8	98.2
48	96.7	~100	99.5	96.3

**Figure A1:**
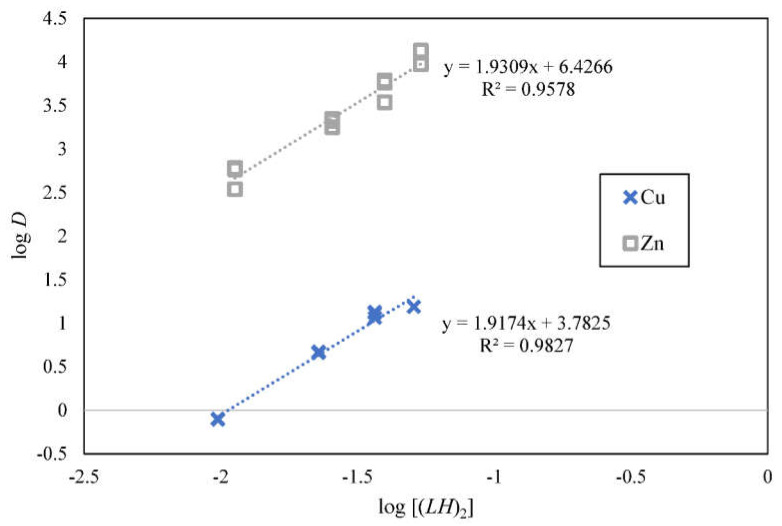
Value of *x* for both copper and zinc were found to be ≈2.

## Data Availability

The authors confirm that the data supporting the findings of this research are available within the article.

## Appendix A

Value of *x* and extraction equilibrium constant, *K_ex_* of copper and zinc can be determined from equilibrium slope analysis and the intercepts of the plots (Figure A1), respectively. Equation for the equilibrium plot was derived from the extraction equilibrium constant expression as follows:

$$K_{ex}=\frac{\left[ML_{2}{\left(LH\right)}_{x}\right]{\left[H^{+}\right]}^{2}}{\left[M^{2+}\right]{\left[{\left(LH\right)}_{2}\right]}^{\frac{2+x}{2}}}$$

$$\log K_{ex}=\log\frac{\left[ML_{2}{\left(LH\right)}_{x}\right]}{\left[M^{2+}\right]}+\log{\left[H^{+}\right]}^{2}-\log{\left[{\left(LH\right)}_{2}\right]}^{\frac{2+x}{2}}$$

whereby [*ML*_2_(*LH*)*_x_*] is the concentration of metal ions extracted into the organic phase, [*M*^2+^] is the concentration of metal ions that remain unextracted in aqueous feed and [(*LH)*_2_] is the concentration of free dimeric D2EHPA that is not bounded to any metal ions at equilibrium. Given that distribution coefficient, $D=\frac{\left[ML_{2}{\left(LH\right)}_{x}\right]}{\left[M^{2+}\right]}$ and $pH=-\log\left[H^{+}\right]$, the equation above can be rewritten as:

$$\log D=\frac{2+x}{2}\log\left[{\left(LH\right)}_{2}\right]+\log K_{ex}+2pH$$

By varying the concentration of D2EHPA for extraction while holding the extraction pH_eq_ constant, value of *x* can be calculated from the slope of the plot of log *D* against log [(*LH)*_2_] whereas *K_ex_* can be calculated from its intercept = $\log K_{ex}+2pH$.

Extraction pH_eq_ was maintained at 3.60, hence:

$$\log K_{ex,Cu}+2pH=3.7825$$

$$K_{ex,Cu}=3.82\times 10^{-4}$$

$$\log K_{ex,Zn}+2pH=6.4266$$

$$K_{ex,Zn}=0.169$$
